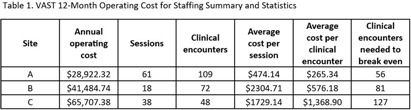# Budget Impact Analysis for the Spread and Financial Sustainability of Videoconference Antimicrobial Stewardship Programs

**DOI:** 10.1017/ash.2024.159

**Published:** 2024-09-16

**Authors:** Amanda Vivo, Dustin French, Corinne Kowal, Oteshia Hicks, Taissa Bej, Brigid Wilson, Sunah Song, Geneva Wilson, Alexandria Nguyen, Amelia Milner, Sara Abdelrahim, Robin Jump, Charlesnika Evans

**Affiliations:** Edward Hines, Jr. VA Hospital; Northwestern University and Hines VAMC; Department of Veteran Affairs; Louis Stokes Cleveland VA Medical Center; VA Northeast Ohio Healthcare System; Institute for Computational Biology; VA Cleveland Healthcare System; VA Pittsburgh Healthcare System; Northwestern University, Feinberg School of Medicine

## Abstract

**Background:** In rural areas, antimicrobial stewardship programs often have limited access to infectious disease (ID) expertise. Videoconference Antimicrobial Stewardship Teams (VASTs) pair rural Veterans Affairs (VA) medical centers with an ID expert to discuss treatment of patients with concerns for infection. In a pilot study, VASTs were effective at improving antimicrobial use. Here, we evaluated 12-month operating costs for staffing for 3 VASTs. **Methods:** We used the following data to describe 12 months of clinical encounters for 3 VASTs operating from January 2022 – March 2023: the number of VAST sessions completed and clinical encounters; Current Procedural Terminology (CPT) codes associated with clinical encounters; session attendees (by role) and the time spent (percent effort) on VAST-related activities. The annual operating cost was based on the annual salaries and percent effort of VAST attendees. We used these characteristics combined with private-sector and Medicare reimbursements to evaluate the cost of implementation and number of clinical encounters needed to offset those costs (breakeven) for each site. **Results:** Three VASTs recorded 229 clinical encounters during 117 sessions (Table 1). Based on CPT codes, the approximate revenue per patient was $516.46. Site A, the only site to break even, had the most sessions and clinical encounters as well as the lowest operating costs. For Site B, a slight increase in the clinical encounters, which might be achieved by 3 additional VAST sessions, would help achieve breakeven. For Site C, increasing the number of clinical encounters to 3-4 per session would have helped their VAST break even without requiring a decrease in operating costs. **Conclusions:** The frequency of VAST sessions, volume of clinical encounters, and low operating costs all contributed the VAST at Site A achieving a financial break-even point within 12 months. Consideration of the potential number of clinical encounters and sessions will help other VASTs achieve financial sustainment, independent of cost-savings related to potential decreases in expenditures for antibiotics and antibiotic-related adverse events. These results also provide insight into possible adoption and diffusion of VAST-like programs in the Medicare hospital setting.

**Figure:**